# Blockade of the Sigma-1 Receptor Relieves Cognitive and Emotional Impairments Associated to Chronic Osteoarthritis Pain

**DOI:** 10.3389/fphar.2019.00468

**Published:** 2019-05-03

**Authors:** Mireia Carcolé, Daniel Zamanillo, Manuel Merlos, Begoña Fernández-Pastor, David Cabañero, Rafael Maldonado

**Affiliations:** ^1^Neuropharmacology Laboratory, Department of Experimental and Health Sciences, Pompeu Fabra University, Barcelona, Spain; ^2^Drug Discovery and Preclinical Development, Laboratories Esteve, Barcelona Science Park, Barcelona, Spain

**Keywords:** osteoarthritis, pain, sigma-1 receptor, cognition, depression, microglia, medial prefrontal cortex

## Abstract

Osteoarthritis is the most common musculoskeletal disease worldwide, often characterized by degradation of the articular cartilage, chronic joint pain and disability. Cognitive dysfunction, anxiety and depression are common comorbidities that impact the quality of life of these patients. In this study, we evaluated the involvement of sigma-1 receptor (σ1R) on the nociceptive, cognitive and emotional alterations associated with chronic osteoarthritis pain. Monosodium iodoacetate (MIA) was injected into the knee of Swiss-albino CD1 mice to induce osteoarthritis pain, which then received a repeated treatment with the σ1R antagonist E-52862 or its vehicle. Nociceptive responses and motor performance were assessed with the von Frey and the Catwalk gait tests. Cognitive alterations were evaluated using the novel object recognition task, anxiety-like behavior with the elevated plus maze and the zero-maze tests, whereas depressive-like responses were determined using the forced swimming test. We also studied the local effect of the σ1R antagonist on cartilage degradation, and its central effects on microglial reactivity in the medial prefrontal cortex. MIA induced mechanical allodynia and gait abnormalities that were prevented by the chronic treatment with the σ1R antagonist. E-52862 also reduced the memory impairment and the depressive-like behavior associated to osteoarthritis pain. Interestingly, the effect of E-52862 on depressive-like behavior was not accompanied by a modification of anxiety-like behavior. The pain-relieving effects of the σ1R antagonist were not due to a local effect on the articular cartilage, since E-52862 treatment did not modify the histological alterations of the knee joints. However, E-52862 induced central effects revealed by a reduction of the cortical microgliosis observed in mice with osteoarthritis pain. These findings show that σ1R antagonism inhibits mechanical hypersensitivity, cognitive deficits and depressive-like states associated with osteoarthritis pain in mice. These effects are associated with central modulation of glial activity but are unrelated to changes in cartilage degradation. Therefore, targeting the σ1R with E-52862 represents a promising pharmacological approach with effects on multiple aspects of chronic osteoarthritis pain that may go beyond the strict inhibition of nociception.

## Introduction

Osteoarthritis is one of the most prevalent chronic diseases and represents a major socio-economic burden worldwide (Johnson and Hunter, 2014; Puig-Junoy and Ruiz Zamora, 2015). It is a complex disease of the whole joint defined by progressive destruction of articular cartilage (Sutton et al., 2009; Zhang et al., 2013). Its most problematic symptoms are pain and loss of joint function, and current pharmacological therapies are limited and generally directed to relief pain. However, osteoarthritis pain is frequently accompanied by co-morbid affective manifestations, such as anxiety and depression (Axford et al., 2010; Goldenberg, 2010; Sharma et al., 2016), and by cognitive alterations including memory dysfunction, which contribute to an overall impairment of the quality of life (Moriarty et al., 2011; Moriarty and Finn, 2014). These co-morbid alterations could in turn aggravate pain perception and contribute to the establishment of chronic osteoarthritis pain (Villemure and Bushnell, 2009). In this context, treatments that simultaneously control the nociceptive, affective and cognitive manifestations could represent an efficient therapeutical approach for chronic osteoarthritis pain.

Sigma-1 receptor (σ1R) is a ligand-regulated chaperone that interacts with a large number of receptors and ion channels (Su and Hayashi, 2003; Hayashi and Su, 2007; Tsai et al., 2009) and has widespread distribution in the nervous system (Harada et al., 1994; Alonso et al., 2000; Kitaichi et al., 2000). Preclinical studies have implicated this receptor in several neurological disorders, such as addiction (Matsumoto et al., 2002; Maurice et al., 2002), schizophrenia (Hayashi et al., 2011) neurodegenerative disorders (Maurice et al., 1998; Francardo et al., 2014) or depression (Urani et al., 2001; Skuza and Rogóz, 2003; Lucas et al., 2008). σ1R has also been proposed as an effective therapeutic target in several models of chronic pain (Entrena et al., 2009; Nieto et al., 2012; Romero et al., 2012; Gris et al., 2014; Tejada et al., 2014). However, these studies do not assess the participation of the σ1R on the emotional or cognitive alterations that can develop after the induction of persistent pain (La Porta et al., 2015, 2016; Negrete et al., 2017). Thus, it remains to be determined whether σ1R ligands could be effective relieving chronic osteoarthritis pain together with its co-morbid cognitive and affective impairments.

The prefrontal cortex plays a crucial role in emotional processing (Gusnard et al., 2001; Etkin et al., 2011), cognitive functions (Phelps et al., 2004) and modulation of pain perception (Apkarian et al., 2004, 2005; Metz et al., 2009). Clinical studies have observed functional and structural abnormalities in the prefrontal cortex of patients suffering from chronic pain (Apkarian et al., 2004; Seminowicz et al., 2011). Such anatomical alterations have also been observed in animal models of neuropathic pain, where a decreased volume of the prefrontal cortex was found in correlation with anxiety-like behavior (Seminowicz et al., 2009). Several studies have also revealed the important role of microglial cells in the adaptative changes occurring in the central nervous system during chronic pain, leading to the persistence of pain manifestations (Racz et al., 2008). The role of microglia on chronic pain has been revealed in the spinal cord (Racz et al., 2008), and supraspinal activation of microglia is also partly responsible for the structural, functional, and molecular neuroplasticity associated with pathological pain (Boadas-Vaello et al., 2017). In fact, it has been proposed that microglial alterations in cortical regions underlie pain-induced emotional and cognitive impairments (Panigada and Gosselin, 2011). Therefore, targeting microglial reactivity in these areas could be an appropriate strategy to treat the affective and memory disturbances observed in chronic pain conditions. Interestingly, σ1R is highly expressed in microglia (Gekker et al., 2006) where it exerts a modulatory function (Peviani et al., 2014; Moritz et al., 2015). Therefore, it would be important to elucidate the possible role of σ1R on cortical microgliosis associated to chronic osteoarthritis pain.

Here we assessed the effect of the σ1R antagonist E-52862, also named S1RA (Romero et al., 2012; Gris et al., 2014) and MR309 (Castany et al., 2018), on the nociceptive, cognitive and emotional alterations observed in the monosodium iodoacetate (MIA) model of osteoarthritis pain in mice. To determine whether E-52862 exerts its effects through a local participation of σ1R on the knee joint, we analyzed levels of cartilage degradation through histological assessment. In addition, we evaluated possible central neuroplastic effects of the σ1R antagonist by determining microglial density and morphology in the medial prefrontal cortex.

## Materials and Methods

### Animals

Swiss albino male mice (Charles River, Lyon, France) 8–12 weeks old were used in all the experiments. Mice weighted 22–24 g at the beginning of the experiments and were housed in groups of 3–4 with free access to water and food. The housing conditions were maintained at 21 ± 1°C and 55 ± 10% relative humidity in a controlled light/dark cycle (light on between 8:00 a.m. and 8:00 p.m.). During the weekly home cage replacement, the nest and an ounce of the old bedding were kept to reduce stress, and it was scheduled for days without any behavioral testing to avoid interferences. Six to 8 animals were used for each experimental group for behavioral testing, and 5–7 animals for the histological scoring, using a total of 53 mice. All experimental procedures and animal husbandry were conducted following the ARRIVE (Animal Research: Reporting *In Vivo* Experiments) guidelines and according to the ethical principles of the International Association for the Study of Pain (I.A.S.P.) for the evaluation of pain in conscious animals (Zimmermann, 1986) and the European Parliament and the Council Directive (2010/63/EU), and were approved by the Animal Care and Use Committees of the PRBB and *Departament de Territori i Habitatge* of Generalitat de Catalunya. All the experiments were performed under blinded conditions.

### Intra-Articular Injection of MIA

Osteoarthritis pain was induced in mice briefly anesthetized with isoflurane (2% v/v) vaporized in oxygen. The joint was shaved and flexed at a 90° angle and 10 μl of MIA (10 mg/mL, Sigma, United Kingdom) dissolved in sterile saline (NaCl 0.9%) were intra-articularly injected with a 30-gauge needle. Control mice received the same volume of sterile saline.

### Nociceptive Behavior

Hypersensitivity to punctate stimuli (von Frey filaments), which will be referred as mechanical allodynia throughout the text, was used as outcome measure of osteoarthritis pain. For this purpose, hind paw withdrawal response to von Frey filament stimulation was assessed (Chaplan et al., 1994). Briefly, animals were placed in Plexiglas cylinders (20 cm high, 9 cm diameter) positioned on a grid surface through which calibrated von Frey filaments (North Coast Medical, United States) were applied following the up-down paradigm, as previously reported (Chaplan et al., 1994). The 0.4-g filament was used first, and the strength of the next filament was decreased or increased according to the response following this sequence 0.07, 0.16, 0.4, 0.6, 1.0, 2.0. The 2.0-g filament was used as a cut-off. The mechanical threshold (in grams) was then calculated with the up-down Excel program (Dixon, 1965). Animals were habituated for 3 consecutive days (2 h per day) to the von Frey environment before the baseline measurements and for 1 h before testing to allow appropriate behavioral immobility. Clear paw withdrawal, shaking or licking was considered as nociceptive response. Both ipsilateral and contralateral hind paws were tested. Only ipsilateral responses are shown, since contralateral sides showed no significant differences.

### Gait Analysis

We used the Catwalk automated gait analysis (Noldus, Netherlands) to assess the effects of osteoarthritis pain on gait (Vrinten and Hamers, 2003; Ferland et al., 2011). Each mouse was placed individually in the Catwalk walkway, which consists of a glass plate (100 cm × 15 cm × 0.6 cm) plus two Plexiglas walls, spaced 5 cm apart. Mice were allowed to walk freely and traverse from one side to the other of the walkway glass plate. The recordings were carried out when the room was completely dark. A pair of infrared beams spaced 90 cm apart were used to detect mouse arrival and to control (start/stop) data acquisition. LED light from an enhanced fluorescent lamp was emitted inside the glass plate and completely internally reflected. Where mice paws made contact with the glass plate, light was reflected down, and the illuminated contact areas were recorded with a high-speed color video camera. The camera was positioned underneath the glass plate connected to a computer that run the Catwalk software 9.1. The software regarded a run as compliant if the animal did not show a maximum speed variation greater than 40%. Three compliant runs (trial) were recorded for each animal and time point. The software automatically labeled all the areas containing pixels above the set thresholds. These areas were identified and assigned to the respective paws. Data were segmented to only take into account sequences with a minimum number of 10 consecutive steps per run and an average speed between 20 and 90 cm/s. Print area (complete surface area contacted by the paw during a stance phase), maximal contact area (maximum area of a paw that comes into contact with the glass plate), swing (duration in sec of no contact of a paw with the glass plate) and duty cycle (duration in sec of contact of a paw with the glass plate as percentage of a whole step cycle) were analyzed. A ratio between right and left hind paws was calculated.

### Cognitive Behavior

Object recognition memory was assessed with the V-maze (Panlab, Barcelona, Spain) to measure cognitive performance, as previously described (Puighermanal et al., 2009; Saravia et al., 2019). V-maze consisted on an apparatus made of black plexiglass with two corridors (30 cm long × 4.5 cm wide) set in V with a 90° angle and 15 cm-high walls. This task consists of 3 sessions of 9 min each (habituation, training and test). On day 1, mice were^1^ habituated to the empty maze. On the 2nd day, mice were put back and 2 identical objects were presented at the end of each of the corridors. Mice were placed again in the maze 24 h later and one of the familiar objects was replaced with a novel object. Time exploring each of the 2 objects (novel and familiar) was recorded. A discrimination index [(time exploring the novel object - time exploring the familiar)/(time exploring novel + familiar) ^∗^ 100] was used as outcome measure of cognitive behavior. High values of discrimination represent good recognition memory. Total time of exploration of the 2 objects was used as a measure of locomotor activity.

### Affective Behavior

The elevated plus maze was used to evaluate anxiety 11 days after saline or MIA injection. It was performed in a black Plexiglas apparatus with 4 arms (29 cm long × 5 cm wide), 2 open and 2 closed, set in cross from a neutral square (5 cm × 5 cm) elevated 30 cm above the floor and indirectly illuminated from the top (40–50 lux in the open arms/4–6 lux in the close arms). 5-min test sessions were performed, and the latency to the first entrance to the open arms and the percentage of entries and time spent in the open arms were used as a measure of anxiety-like behavior (Cruz et al., 1994). Mice were habituated to the testing room for 1 h before starting the evaluation, and the equipment was carefully cleaned between subjects.

The elevated zero maze was used as additional measure of anxiety-like behavior 21 days after saline or MIA injection. Mice were placed in a black Plexiglas apparatus with a round shape, where 2 quarters of the maze were closed by walls (20 cm-high) and elevated 30 cm above the floor. Sessions were 5 min long, and the latency to the open quadrants and the percentage of time spent in the open parts was determined (Shepherd et al., 1994). Mice were habituated to the testing room for 1 h before starting the evaluation, and the equipment was carefully cleaned between subjects.

The forced swimming test was used to evaluate depressive-like behavior 25 days after saline/MIA (Porsolt et al., 1977). Mice were placed for 6 min into transparent Plexiglass cylinders (17.5 cm high and 12.5 cm diameter) filled with 15 cm of water at 22 ± 2°C. The percentage of time of immobility was assessed for the last 4 min. Immobility was considered when the animal made no movements in order to escape (swimming, climbing the walls). Mice were habituated to the testing room for 1 h before starting the evaluation, the equipment was carefully cleaned, and the water was changed between subjects.

### Experimental Protocol

Animals were carefully handled and habituated to the von Frey environment for 3 consecutive days before the baseline measurement. The day following baseline nociceptive assessment, MIA or saline was injected into the knee joint. Mice were intraperitoneally treated twice a day (10 a.m. and 06 p.m.) with either vehicle or E-52862 (20 mg/kg) from the 1st day after the intra-knee injection to the end of the experiment on day 25. Mechanical sensitivity was evaluated 5 and 19 days after the intra-articular injection with the von Frey test, and at days 6 and 12 with the Catwalk gait test. Nociceptive assessments were performed 30 min after drug administration. Cognitive and affective behavior were also analyzed. For this purpose, the elevated plus maze was performed 11 days after intra-knee injection, the novel object recognition task at days 13, 14, and 15 (habituation, training, and test), the zero-maze at day 21 and the forced swimming test at day 25 after MIA/saline. The late anxiety-like behavior was assessed with a different test from the early evaluation to avoid the well-reported one-trial tolerance to the behavioral test (File et al., 1990; Holmes and Rodgers, 1998). The days of the evaluation of affective behavior, E-52862-treated mice received vehicle instead of the σ1R antagonist 30 min before the test to avoid acute effects of the drug, and E-52862 was administered after the test to continue the repeated treatment. An additional group of vehicle-treated mice received a single dose of E-52862 before the novel object recognition task. Tissue for immunofluorescence analysis was extracted on day 26 and 12 ± 1 h after the last drug administration.

### Drugs

The selective σ1R antagonist E-52862 [(4-[5-methyl-1-(2-naphthalenyl)-1H-pyrazol-3-yl]oxy]ethyl] morpholine hydrochloride] was developed and supplied by Laboratories Esteve (Barcelona, Spain). E-52862 was dissolved in an aqueous solution (0.5% hydroxypropylmethyl cellulose, HPMC; Sigma-Aldrich) and administered by intraperitoneal route at a volume of 10 ml/kg 30 min before behavioral testing.

### Histology

#### Knee Joint Isolation

A separate group of mice was intra-knee injected with saline or MIA and intraperitoneally repeatedly treated with vehicle or E-52862. Mice were sacrificed by cervical dislocation 29 days after the experimental induction of osteoarthritis pain. The ipsilateral knee joints were subsequently removed, post-fixed 48 h in 4% paraformaldehyde, and then cryopreserved in 30% sucrose solution at 4°C.

#### Histological Preparation

The fixed knee joints were decalcified in Osteomoll (Merck, Germany) for 6–7 h and left overnight in 30% sucrose solution. The joints were subsequently embedded in gelatine (7.5%) and frozen in cold 2-methyl-butane. Coronal 16- to 18-μm sections were cut in a cryostat from the frontal plane toward the back of each joint and mounted on gelatinized slides (6–7 slides with 10 sections each). All the serial sections were stained with the Safranin O-Fast Green staining protocol. Briefly, after hydrating sections with decreasing concentrations of ethanol, sections were stained with haematoxylin (Merck, Germany) and subsequently with 0.002% Fast Green (Sigma, Spain) and 0.2% Safranin O (Merck, Germany) solutions. The sections were finally dehydrated and cleared with increasing ethanol concentrations and xylene, then mounted with Eukitt (O. Kindler, Germany) and a covering glass. All the stained sections were viewed with a 10× objective using a Leica DMR microscope equipped with a Leica DFC 300 FX digital camera. Nine images of the obtained sections spanning the central load-bearing region of the knee were taken for both medial and lateral sides of each joint (18 total images per joint) and used for histological scoring.

#### Histological Scoring

A semiquantitative scoring system for murine histopathology, the OARSI score (Glasson et al., 2010) was applied and adapted to our experimental conditions (Figure 2C). All 4 quadrants of the knee joint were evaluated: medial femoral condyle (MFC), lateral femoral condyle (LFC), medial tibial plateau (MTP), and lateral tibial plateau (LTP). A score from 0 to 6 was given to each quadrant of 9 serial sections per animal, having a total of 36 values per animal. The final histological scores were expressed as the sum of all the individual values and the average summed score for each experimental group was calculated. The same observer scored all the histological changes and was blinded to the specimen samples.

### Tissue Isolation

On day 26 after osteoarthritis induction, both MIA and saline mice were deeply anesthetized by intraperitoneally injection (0.2 ml/10 g of body weight) of a mixture of ketamine (100 mg/kg) and xylazine (20 mg/kg) prior to intracardiac perfusion of 4% PFA in 0.1 M Na_2_HPO_4_/NaH_2_PO_4_ buffer, pH 7.5, delivered with a peristaltic pump at 22 ml per min for 2 min. Brains were removed and post-fixed overnight at 4°C in the same fixative solution. Then, brains were transferred to a solution of 30% sucrose in PB 0.1 M and kept at 4°C. Coronal brain sections (30 μm) containing the prelimbic and infralimbic prefrontal cortex were obtained with a microtome (Leica) and kept in a solution of 5% sucrose PB 0.1 M at 4°C until processed for immunofluorescence analysis.

### Immunofluorescence

Free-floating slices were rinsed in PB 0.1 M and blocked in a solution containing 3% normal goat serum and 0.3% Triton X-100 in PB 0.1 M during 2 h at room temperature. The slices were incubated overnight at 4°C with the primary antibody anti-Iba-1 (1:500, rabbit, Wako). The next day, after 3 rinses in PB 0.1 M, sections were incubated for 2 h at room temperature with the secondary antibody AlexaFluor-555 goat anti-rabbit (1:1000, Life Technologies). Then, slices were rinsed 3 times and mounted with Fluoromount onto glass slides coated with gelatine.

### Immunofluorescence Image Analysis

The stained sections were analyzed with the 40× objective and 1× zoom using a confocal microscope (Leica TCS SP5 STED). A *z*-stack image of 30 μm with 0.5 depth intervals was obtained from every slice. Density^2^ and cell architecture of microglia was examined using the ImageJ analysis software. The perimeter of microglial soma was measured using the tool “Freehand line” and the option “Analyze and Measure.” Four images per brain area of 6 animals per group were analyzed.

### Statistical Analysis

A 3-way repeated measure analysis of variance (ANOVA) with surgery and treatment as between-subject factors and day as within-subject factor was used to analyze von Frey and gait data. 2-way ANOVA (surgery and treatment) was used to analyze affective behavioral data, as well as the histological scoring from the joint, and 1-way ANOVA was used to analyze the cognitive behavior. In all comparisons, Fisher Least Significant Difference (LSD) *post hoc* analysis was applied when appropriate (significant interaction between factors). STATISTICA 6.0 (StatSoft, Inc., Tulsa, OK, United States) software was used. The differences were considered statistically significant when the *P*-value was below 0.05 (Supplementary Table S1).

## Results

### The σ1R Antagonist E-52862 Reverses Mechanical Hypersensitivity Associated to Osteoarthritis Pain

**FIGURE 1:**
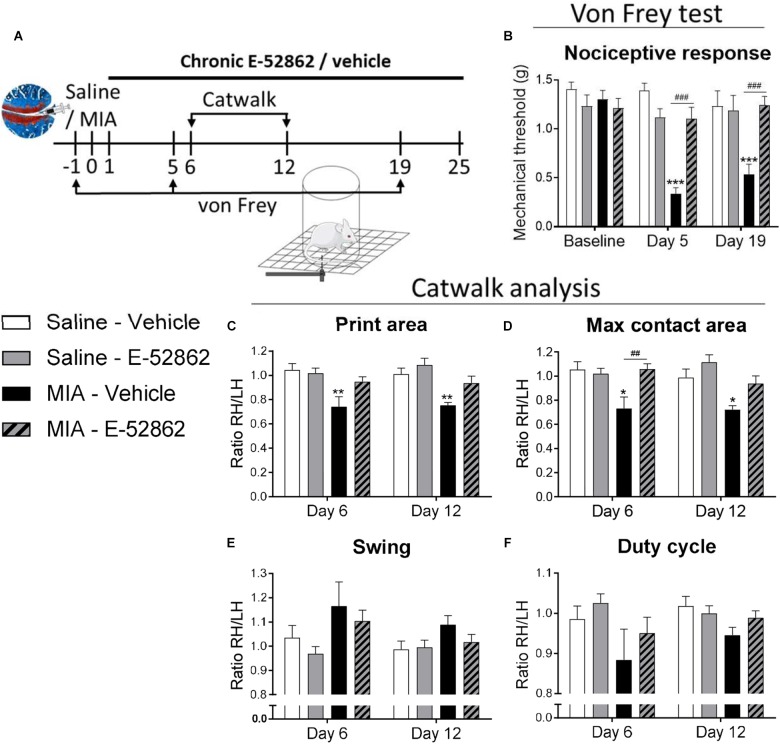
Repeated treatment with E-52862 reversed mechanical hypersensitivity associated to osteoarthritis pain. **(A)** Mice received an intra-knee injection of MIA or saline and were treated with vehicle or E-52862 (20 mg/kg) from day 1 until the end of the experiment (day 25). Mechanical thresholds were assessed with the von Frey under basal conditions and on days 5 and 19 after the intra-articular injection, and gait was analyzed with the Catwalk test on days 6 and 12. **(B)** MIA-injected mice treated with vehicle showed a decrease on mechanical thresholds that was reversed in E-52862-treated mice. Catwalk analysis revealed a decrease of the ratio (right hind/left hind paws) of print area **(C)** and maximal contact area **(D)** in mice injected with MIA and treated with vehicle. This alteration was reversed in mice receiving E-52862. Swing **(E)** and duty cycle **(F)** were not significantly altered by the intra-knee injection. Data are expressed as mean ± SEM (*n* = 6–8 animals per group). ^∗^*p* < 0.05, ^∗∗^*p* < 0.01, ^∗∗∗^*p* < 0.001 vs. Saline-vehicle, ^##^*p* < 0.01, ^###^*p* < 0.001 for MIA-vehicle vs. MIA-E-52862 (3-way repeated measures ANOVA followed by Fisher Least Significant Difference test). MIA, monosodium iodoacetate; SEM, standard error of the mean; RH, right hind; LH, left hind.

To evaluate the effect of E-52862 on mechanical hypersensitivity associated to osteoarthritis pain, mice were intraperitoneally treated twice a day with either vehicle or E-52862 (20 mg/kg) from the 1st day after MIA injection until the end of the experiment (day 25). Von Frey test was performed before and 5 and 19 days after MIA injection, and gait analysis was evaluated at 6 and 12 days (Figure 1A). MIA injection induced a persistent mechanical hypersensitivity in vehicle-treated mice (*p* < 0.001 vs. saline mice, days 5 and 19). Conversely, this decrease in mechanical thresholds was absent in mice treated with E-52862 (*p* < 0.001 vs. MIA vehicle mice, days 5 and 19) (Figure 1B). Gait analysis also showed MIA-induced alterations on walking patterns that were partly reversed by E-52862. Mice injected with MIA and treated with vehicle showed a significant decrease of the print area (*p* < 0.01 vs. saline; Figure 1C) and maximal contact area (*p* < 0.05 vs. saline; Figure 1D) at both time points tested. These alterations were not observed when MIA-injected mice were treated with E-52862 (Figure 1C,D). No significant effects were observed in the swing for any of the experimental groups (Figure 1E), however, a trend toward a decreased duty cycle was observed in MIA mice treated with vehicle (*p* = 0.08 vs. saline; Figure 1F). Therefore, blocking the σ1R produced a relief of mechanical pain associated to the injection of MIA that was also reflected into a normalization of gait function.

### MIA Injection Into the Knee Produces Cartilage Degradation Insensitive to the σ1R Antagonist E-52862

**FIGURE 2:**
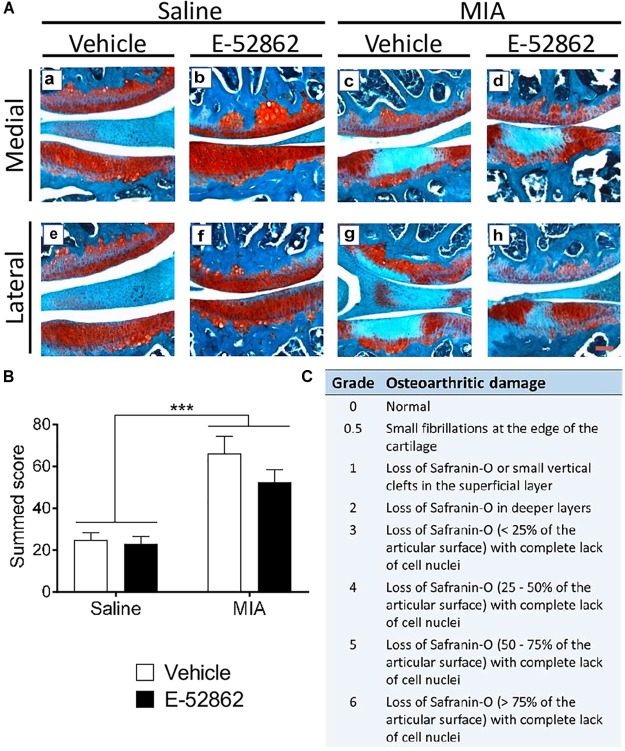
Histological knee alterations in mice injected with MIA were not prevented by the chronic treatment with E-52862. Ipsilateral knees of saline and MIA mice were obtained 29 days after intra-articular injection in mice receiving vehicle or E-52862 treatment. **(A)** Medial and lateral sides of the joints are represented, showing the femur condyle (above) and the tibial plateau (below). **(B)** The injection of MIA produced cartilage degeneration revealed by an increased OARSI score. Treatment with E-52862 (20 mg/kg, twice daily during 25 days) did not prevent the joint damage. **(C)** The semiquantitative scoring system for joint histopathology. The scores for each image are (first value represents femur condyle and second value represents tibial plateau): **(a)** 1, 0.5; **(b)** 0.5, 0; **(c)** 2, 5; **(d)** 2, 5; **(e)** 2, 0.5; **(f)** 0, 0.5; **(g)** 3, 6; **(h)** 2, 3. Data are expressed as the mean ± SEM (*n* = 5–7 animals per group). Scale bar: 100 μm. ^∗∗∗^*p* < 0.001 for saline vs. MIA (2-way ANOVA). MIA, monosodium iodoacetate; SEM, standard error of the mean.

Monosodium iodoacetate is a chondrocyte glycolytic inhibitor which produces chondrocyte death and damage in the entire joint space. We determined the level of cartilage degeneration through proteoglycan staining 29 days after the intra-knee injection (Figure 2A). MIA injected mice had a clear increase on the OARSI score when compared to saline mice (*p* < 0.001; Figure 2B), and no significant effect of the E-52862 treatment (20 mg/kg, twice daily during 25 days) was found. Therefore, joint damage was not significantly prevented by the blockade of σ1R.

### Acute and Chronic Blockade of σ1R Avoid Osteoarthritis-Induced Cognitive Impairment

**FIGURE 3:**
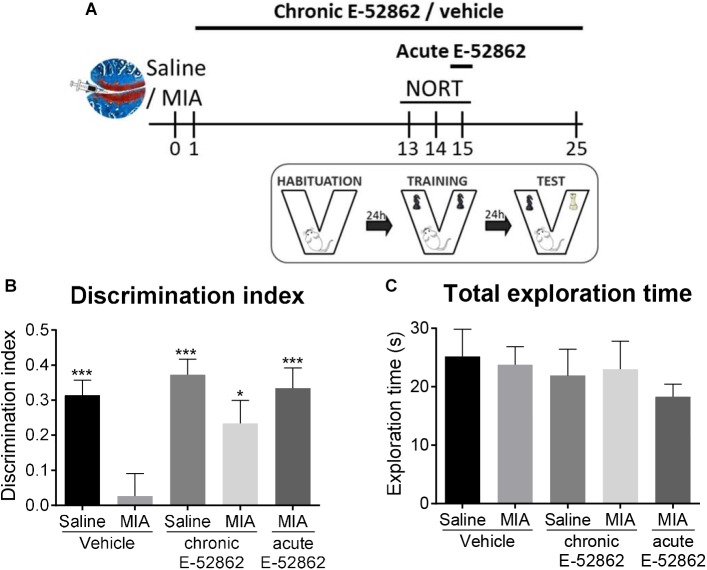
Acute and chronic treatments with E-52862 improved the cognitive deficits induced by MIA injection. **(A)** Saline or MIA-injected mice treated with vehicle or E-52862 (20 mg/kg, twice daily during 25 days) were evaluated for recognition memory 15 days after the intra-knee injection in the novel object recognition test (NORT). **(B)** Mice with osteoarthritis pain treated with vehicle showed decreased discrimination index indicating a memory impairment. Acute and chronic treatment with E-52862 reversed the cognitive deficits induced by MIA. **(C)** Animals revealed similar total exploration times regardless of the surgery or the treatment. Data are expressed as mean ± SEM (*n* = 6–8 animals per group). ^∗^*p* < 0.05, ^∗∗∗^*p* < 0.001 vs. MIA-vehicle (1-way ANOVA followed by Fisher least significant difference test). MIA, monosodium iodoacetate; SEM, standard error of the mean.

Chronic pain is often accompanied by memory dysfunction. Therefore, we analyzed the effect of chronic treatment with E-52862 (20 mg/kg, twice daily during 25 days) over recognition memory in the osteoarthritis model (Figure 3A). The novel object recognition task performed 15 days after MIA/saline injection showed a significant decrease on the discrimination index of MIA-injected mice treated with vehicle (*p* < 0.001 vs. saline). This cognitive impairment was avoided after the chronic treatment with E-52862 (*p* < 0.05 vs. MIA vehicle; Figure 3B). Interestingly, MIA-injected mice receiving a single acute dose of the σ1R antagonist (20 mg/kg) 30 min before the test also showed an improvement on the discrimination index (*p* < 0.001 vs. MIA vehicle) (Figure 3B). All groups of mice showed similar total exploration times, suggesting normal locomotor activity in this paradigm regardless of the surgery or the treatments (Figure 3C). Therefore, the impairment of recognition memory caused by chronic osteoarthritis pain was improved after chronic or acute blockade of σ1R.

### E-52862 Decreases Depressive-Like Behaviour Associated to Osteoarthritis Pain

**FIGURE 4:**
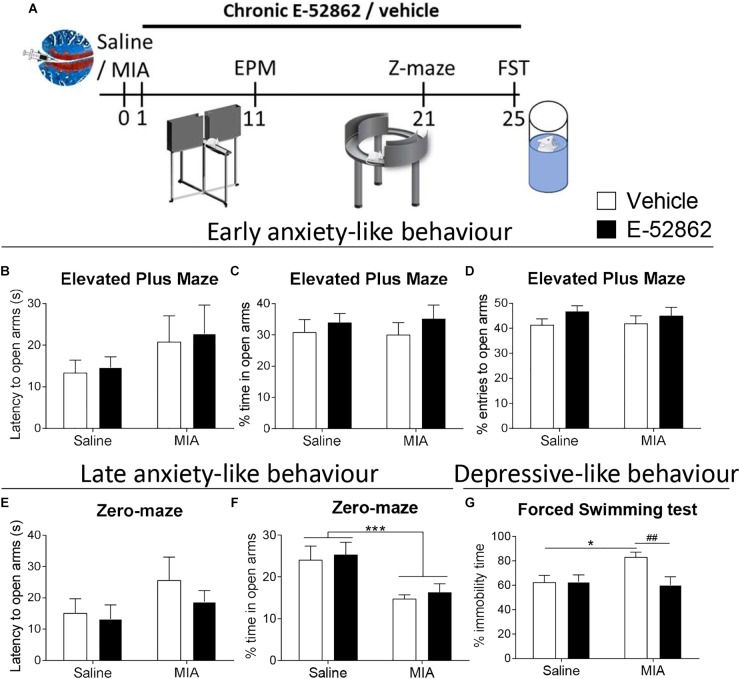
E-52862 treatment reduced depressive-like behavior, but not anxiety-like responses associated with chronic osteoarthritis pain. **(A)** Emotional manifestations of osteoarthritis pain were assessed in saline- or MIA-injected mice after repeated administration with vehicle or E-52862 (20 mg/kg, twice daily during 25 days) to saline or MIA-injected mice. Anxiety-like behavior was evaluated on day 11 after the intra-knee injection with the elevated plus maze (EPM), and at day 21 in the zero-maze (Z-maze), while depressive-like behavior was determined in the forced-swimming test (FST) on day 25. The latency to enter in the open arms **(B)**, and the percentage of time **(C)** and entries **(D)** to the open arms of the EPM showed no significant differences between groups. At day 21, no significant differences were observed in the latency to the open quadrants of the zero-maze **(E)**, whereas mice injected with MIA and treated with vehicle spent less time in the open parts **(F)**. This increase on late anxiety-like behavior was not modified by E-52862 treatment. **(G)** Mice with osteoarthritis pain receiving vehicle showed increased immobility time, which was reversed by E-52862 treatment. Data are expressed as mean ± SEM (*n* = 6–8 animals per group). For **(D)**: ^∗∗∗^*p* < 0.001 for saline vs. MIA (two-way ANOVA). For **(E)**: ^∗^*p* < 0.05 for saline – vehicle vs. MIA – vehicle, ^##^*p* < 0.01 for MIA – vehicle vs. MIA – E-52862 (two-way ANOVA). MIA, monosodium iodoacetate; SEM, standard error of the mean.

Anxiety and depressive-like behavior were assessed to determine whether E-52862 (20 mg/kg, twice daily during 25 days) could modulate emotional-like states associated to osteoarthritis pain (Figure 4A). It has been proposed that the initial stages of osteoarthritis pain are associated with inflammatory processes, whereas later stages involve neuropathic components, which may differentially affect the emotional manifestations. Thus, early and late anxiety-like behavior was assessed in our model. Early anxiety was evaluated 11 days after intra-knee injection in the elevated plus maze. No differences were found between saline- and MIA-injected mice in the latency to entry to the open arms, and the percentage of time and entries to the open arms, regardless of the treatment received (Figure 4B–D). On the other hand, despite the latency to the open quadrants of the zero-maze was not altered (Figure 4E), mice with osteoarthritis pain showed late anxiety-like behavior reflected in a significant decrease of the time spent in the open arms of the zero-maze (*p* < 0.001 vs. saline), also regardless of the treatment. Thus, E-52862 did not normalize the anxiogenic-like responses induced by MIA (Figure 4F). Depressive-like behavior was analyzed in the forced swimming test 25 days after the intra-articular injection. In this paradigm, mice with osteoarthritis pain receiving vehicle showed a significant increase on immobility time (*p* < 0.05 vs. saline; Figure 4G). Chronic E-52862 administration prevented such an increase in despair-like behavior (*p* < 0.01 vs. MIA vehicle; Figure 4G). Therefore, anxiety-like behavior was not sensitive to σ1R antagonism, whereas MIA-induced depressive-like behavior was prevented after E-52862 treatment.

### E-52862 Modulates Microglial Expression in the Medial Prefrontal Cortex

**FIGURE 5:**
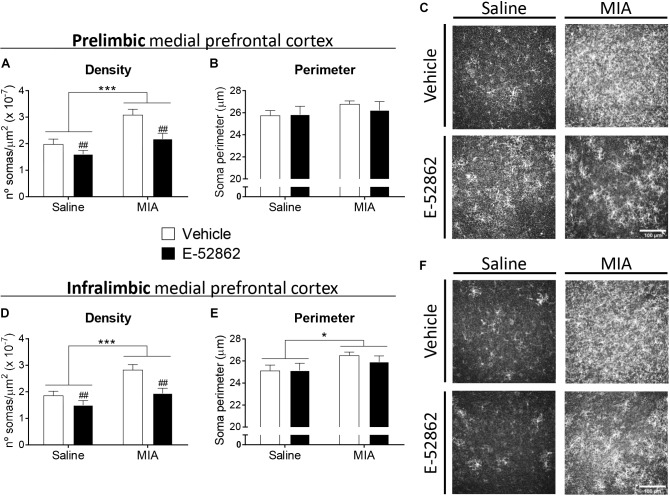
E-52862 decreased pain-induced microgliosis in the medial prefrontal cortex. Microglial cells were detected in the prelimbic and infralimbic areas of the medial prefrontal cortex (mPFC) of saline and MIA injected mice after the repeated treatment with vehicle or the σ1R antagonist E-52862 (20 mg/kg, twice daily during 25 days). Mice with osteoarthritis pain treated with vehicle showed an increased density of microglial cells in the prelimbic **(A)** and the infralimbic areas **(D)**. This microgliosis was normalized by the treatment with E-52862 **(A,D)**. MIA-injected mice revealed larger perimeters of the soma of microglial cells in the infralimbic **(E)**, but not in the prelimbic area **(B)**. Treatment with E-52862 did not modify this alteration **(E)**. **(C,F)** Representative images of all groups are shown. Data are expressed as the mean ± SEM (*n* = 7 animals per group). Scale bar: 100 μm. ^∗^*p* < 0.05, ^∗∗∗^*p* < 0.001 for saline vs. MIA, ^##^*p* < 0.01 for vehicle vs. E-52862 (two-way ANOVA). MIA, monosodium iodoacetate; SEM, standard error of the mean.

A possible central role of σ1R modulating microglial activity was assessed through quantification of the density of microglial cells and the perimeter of the somas in the prelimbic and infralimbic areas of the medial prefrontal cortex (Figure 5). The analysis of the cellular density showed a significant increase on the total number of microglial cells in the prelimbic and the infralimbic areas of mice with osteoarthritis pain receiving vehicle (*p* < 0.001 vs. saline) (Figure 5A,C,D,F). Repeated administration of the σ1R antagonist (20 mg/kg, twice daily during 25 days) significantly reduced the microglial density in both cortical areas (*p* < 0.01 vs. MIA vehicle; Figure 5A,C,D,F). MIA-injected mice had an increase of the perimeter of microglial cells in the infralimbic (*p* < 0.05; Figure 5E), but not in the prelimbic area (Figure 5B) when compared to saline-injected mice. This increase was not significantly affected by the treatment with E-52862. Therefore, E-52862 modulated the density of microglial cells in the medial prefrontal cortices without affecting microglia activation.

## Discussion

The present study reveals the involvement of the σ1R in the nociceptive, emotional and cognitive alterations associated with osteoarthritis pain in mice. Mechanical allodynia and gait impairments induced by MIA injection were partly prevented by chronic administration of the σ1R antagonist E-52862. This treatment also inhibited the cognitive deficits and depressive-like behavior of mice with osteoarthritis pain, although anxiogenic-like responses were not modified. Modulation of the pain-induced behavioral alterations by E-52862 was not due to an inhibition of joint damage produced by MIA, and there was a concomitant decrease on MIA-induced microgliosis in the medial prefrontal cortex.

σ1R is highly expressed in key areas for pain control (Alonso et al., 2000; Bangaru et al., 2013). Behavioral studies have shown analgesic efficacy of the σ1R antagonist E-52862 in acute (Romero et al., 2012; Gris et al., 2014; Tejada et al., 2014) and chronic (Gris et al., 2014) models of inflammatory pain, and in neuropathic pain models induced by partial sciatic nerve ligation (Romero et al., 2012), chemotherapy (Nieto et al., 2012), or streptozotocin-induced diabetes (Gris et al., 2016). However, the role of σ1R has not been previously assessed in models of osteoarthritis pain, one of the most prevalent and disabling chronic pain conditions. We showed that E-52862 inhibited both mechanical hypersensitivity and gait alterations in the MIA model of osteoarthritis pain. Gait alterations could be associated to structural modifications of the joint or to the increased mechanical sensitivity (Boettger et al., 2009). Previous studies using the antigen-induced arthritis model in rats suggested that specific gait parameters, such as the angle between the paws, were exclusively influenced by the structural damage of the joint as indicated by its correlation with cartilage destruction (Boettger et al., 2009). However, other parameters, such as the paw print area, represent good measures of pain (Boettger et al., 2009). The correlation with mechanical allodynia would be in agreement with previous work showing that nerve-injured rats with decreased mechanical thresholds to punctate stimulation had also altered walking patterns (Vrinten and Hamers, 2003). In the same line, the MIA model of osteoarthritis pain in rodents showed that celecoxib and morphine reduced mechanical allodynia and gait abnormalities (Ferland et al., 2011; Ferreira-Gomes et al., 2012), suggesting that both parameters are associated in this chronic pain model. Such correlation has also been described in higher order mammals with osteoarthritis pain (Haussler et al., 2007; Frost-Christensen et al., 2008; Moreau et al., 2011; Cake et al., 2013). Thus, the reduction of the paw print area and the maximal contact area parameters observed in our study in osteoarthritic mice were probably a consequence of an unwillingness of the animal to bear weight on the injured limb, while the normalization of such parameters after E-52862 treatment might be related to reduced pain perception. In agreement, the effect of E-52862 on the walking patterns of mice with osteoarthritis was not accompanied by a normalization of the structural alterations observed in the histological assessments. This absence of effect on cartilage damage is in agreement with the low expression levels of σ1R in chondrocytes and bone marrow when compared to its expression in the peripheral and central nervous system^1,2^. The relief of mechanical hypersensitivity and pain-associated comorbidities after the treatment with E-52862 coexisted with the cartilage degradation, in agreement with the widely recognized fact that the presence and severity of joint pain poorly correlates with structural joint damage in osteoarthritis patients (Lawrence et al., 1966; Dieppe, 2004). Thus, the pain-relieving effects of the σ1R antagonist probably rely on a modulatory role on the nervous system and are independent of the site of the primary lesion.

We observed a cognitive deficit associated to osteoarthritis induced by MIA, which was significantly reduced by the repeated administration of E-52862. Our result suggests that the blockade of σ1R plays a protective role in this long-term memory impairment produced by chronic pain. Previous studies also showed impaired memory function in other chronic pain models (Zhao et al., 2006; Kodama et al., 2011) and specifically during MIA-induced joint pain (La Porta et al., 2015; Negrete et al., 2017). Selective σ1R ligands failed to modify learning, consolidation or retention phases of the mnemonic process when administered to naïve animals (Hashimoto et al., 2007; Antonini et al., 2011), but σ1R activation reduced cognitive deficits associated with schizophrenia (Hashimoto et al., 2007), Alzheimer disease (Maurice et al., 1998; Antonini et al., 2011) or scopolamine treatment (Hiramatsu et al., 2002). In contrast, we observed that σ1R blockade reversed the memory impairment induced after MIA injection. The overlap between the neuroanatomical substrates implicated in both pain control and cognitive functions provides information about the development of memory deficits in patients with chronic pain (Moriarty et al., 2011). However, the precise causal mechanisms underlying the pain-related cognitive impairment are still unclear, and the role of the σ1R on this specific type of memory deficits has not been studied. Our data suggest that σ1R antagonists are efficient improving cognitive functions under a chronic pain state.

We obtained increased anxiety-like responses after the intra-knee injection of MIA, as previously reported in other murine models of inflammatory (Schellinck et al., 2003; Chen et al., 2013) and neuropathic pain (Benbouzid et al., 2008; Matsuzawa-Yanagida et al., 2008; La Porta et al., 2016). Anxiety-like behavior was present 3 weeks after MIA, but not at earlier time points (11 days). Previous studies suggested that persistent pain may trigger alterations in brain areas involved in affective responses, which over time may lead to emotional comorbidities including anxiety and depressive-like behavior (Narita et al., 2006; Suzuki et al., 2007; Seminowicz et al., 2009; Sellmeijer et al., 2018). In agreement, 25 days after the intra-knee injection of MIA depressive-like responses were observed in animals with osteoarthritis pain, as in previous studies investigating inflammatory and neuropathic pain (Hasnie et al., 2007; Suzuki et al., 2007; Norman et al., 2010; Negrete et al., 2017). Depressive-like responses were abolished after chronic administration of E-52862, although anxiety-like behavior was not modified with this σ1R antagonist. These results are in line with previous works studying affective behavior in σ1R knockout mice. In these studies, σ1R knockouts exhibited increased immobility in the forced swimming test, but normal anxiety-like behavior (Sabino et al., 2009), suggesting distinct roles of the receptor modulating depressive and anxiety responses. Common neuroplastic changes associated with chronic pain and emotional disorders were proposed as important routes for the onset and reciprocal aggravation of both pathologies (Sheng et al., 2017). Consequently, analgesic drugs such as opioids (Mague et al., 2003; Tenore, 2008) or benzodiazepines (Vollenweider et al., 2011) have been proposed as a treatment for chronic pain-induced depression, and antidepressants like selective serotonin reuptake inhibitors (SSRIs) (Tasmuth et al., 2002; Gebhardt et al., 2016) or tricyclic antidepressants (Rowbotham et al., 2005; Kopsky and Keppel Hesselink, 2012) exhibited antinociceptive effects under chronic pain conditions. The interest of σ1R ligands for the treatment of depressive states raised from the observation that several antidepressants had moderate to high affinity for σ1R sites (Schmidt et al., 1989; Itzhak et al., 1991; Narita et al., 1996). While some SSRIs such as fluvoxamine or venlafaxine have shown agonism for σ1R, others like sertraline may act as antagonists (Ishima et al., 2014). Moreover, the antidepressant efficacy of σ1R ligands may depend on the affective status of the animal, since the selective σ1R agonist PRE-084 reduced depressive-like behavior in adrenalectomized mice but lacked effect in naïve animals (Urani et al., 2001).

We observed an increased microgliosis in the medial prefrontal cortex produced by the injection of MIA. This result agrees with a previous study showing increases of microglial density in the infralimbic cortex of nerve-injured rats (Chu Sin Chung et al., 2017; Xu et al., 2017). Other brain areas such as the amygdala, periaqueductal gray (PAG) or hippocampus, have also shown increased gliosis during chronic pain conditions (Humo et al., 2019). Interestingly, a recent study on neuropathic pain showed enhanced expression of microglial markers in the prefrontal cortex accompanied by depressive-like behavior. Chronic minocycline attenuated both microglial activation and depressive-like responses (Xu et al., 2017). Previous studies have shown that the σ1R antagonist BD1047 attenuated microglial activation in the spinal cord in a model of bone cancer pain (Zhu et al., 2015), but the effect of σ1R on supraspinal microglia has not been assessed in chronic pain models. Our data show that σ1R antagonist E-52862 significantly reduced the density of microglia in medial prefrontal cortices of mice with osteoarthritis pain. This effect was not accompanied by a reduction of anxiety-like behavior, suggesting that this affective disturbance is not directly related to cortical microgliosis. However, these anatomical changes correlated with the cognitive performance and the depressive-like behavior, pointing toward an involvement of cortical microglia on both pain comorbidities. Therefore, σ1R-regulated cortical microgliosis might be crucial for the manifestation of cognitive and emotional alterations often present in chronic pain conditions. Indeed, antidepressant drugs such as SSRIs also have activity modulating microgliosis and reducing microglial production of tumor necrosis factor α and nitric oxide (Chung et al., 2011; Tynan et al., 2012). It is well known that σ1R modulates several signal transduction pathways, including the production of ATP, reactive oxygen species or mitogen-activated protein kinases (MAPK) (Zamanillo et al., 2013; Hayashi, 2015; Zhao et al., 2017). All these molecules have been identified as effective signals for microglial migration and activation (Biber et al., 2007; Fan et al., 2017), suggesting an indirect modulatory role of σ1R. In agreement, σ1R activation by methamphetamine induces a microgliosis that involves generation of reactive oxygen species and activation of the MAPK pathway (Chao et al., 2017).

The present study reveals that E-52862 alleviates the nociceptive, cognitive and emotional manifestations associated to chronic osteoarthritis pain. We provide evidence showing that the effect of σ1R over these manifestations of chronic pain is not associated to local changes in articular damage but is accompanied by modulation of microglial activity in the medial prefrontal cortex. Our data highlight the blockade of σ1R as an interesting pharmacological strategy for the simultaneous management of multiple aspects of chronic osteoarthritis pain.

## Ethics Statement

All experimental procedures and animal husbandry were conducted following the ARRIVE (Animal Research: Reporting *In Vivo* Experiments) guidelines and according to the ethical principles of the International Association for the Study of Pain (I.A.S.P.) for the evaluation of pain in conscious animals (Zimmermann, 1986) and the European Parliament and the Council Directive (2010/63/EU), and were approved by the Animal Care and Use Committees of the PRBB and Departament de Territori i Habitatge of Generalitat de Catalunya.

## Author Contributions

All authors listed have made a substantial, direct and intellectual contribution to the work, and approved it for publication.

## Conflict of Interest Statement

The authors declare that the research was conducted in the absence of any commercial or financial relationships that could be construed as a potential conflict of interest. The handling Editor declared a past co-authorship with several of the authors.

## Footnotes

1 https://www.ebi.ac.uk/gxa/genes/ENSG00000147955?bs=%7B%22homo%20sapiens%22%3A%5B%22ORGANISM_PART%22%5D%7D&ds=%7B%22kingdom%22%3A%5B%22animals%22%5D%7D#baseline

2 https://www.proteinatlas.org/ENSG00000147955-SIGMAR1/tissue
